# Pancreatic neuroendocrine tumor with stenosis of the main pancreatic duct leading to pancreatic pleural effusion: a case report

**DOI:** 10.1186/s40792-020-00987-7

**Published:** 2020-09-25

**Authors:** Yuta Yoshida, Ippei Matsumoto, Tomonori Tanaka, Kentaro Yamao, Akihiro Hayashi, Keiko Kamei, Shumpei Satoi, Atsushi Takebe, Takuya Nakai, Mamoru Takenaka, Yoshifumi Takeyama

**Affiliations:** 1 Department of Surgery, Kindai University Faculty of Medicine, 377-2 Ohno-higashi, Osaka-sayama, Osaka 589-8511 Japan; 2 Department of Pathology, Kindai University Faculty of Medicine, 377-2 Ohno-higashi, Osaka-sayama, Osaka 589-8511 Japan; 3 Department of Gastroenterology and Hepatology, Kindai University Faculty of Medicine, 377-2 Ohno-higashi, Osaka-sayama, Osaka 589-8511 Japan; 4 Gastroenterology, Tottori Prefectural Central Hospital, 730, Ezu, Tottori, Tottori 680-0000 Japan

**Keywords:** Pancreatic neuroendocrine tumor, Pancreatic pleural effusion, Pancreatic ascites, Internal pancreatic fistula, Stenosis of the main pancreatic duct, Pseudocyst in the pancreatic tail

## Abstract

**Background:**

Pancreatic pleural effusion and ascites are defined as fluid accumulation in the thoracic and abdominal cavity, respectively, due to direct leakage of the pancreatic juice. They usually occur in patients with acute or chronic pancreatitis but are rarely associated with pancreatic neoplasm. We present here an extremely rare case of pancreatic neuroendocrine tumor with stenosis of the main pancreatic duct, leading to pancreatic pleural effusion.

**Case presentation:**

A 51-year-old man complained of dyspnea. Left-sided pleural effusion was detected on the chest X-ray. Pleural puncture was performed, and the pleural fluid indicated a high amylase content (36,854 IU/L). Hence, the patient was diagnosed with pancreatic pleural effusion. Although no tumor was detected, the computed tomography (CT) scan showed a pseudocyst and dilation of the main pancreatic duct in the pancreatic tail. Magnetic resonance cholangiopancreatography showed a fistula from the pseudocyst into the left thoracic cavity. Endoscopic retrograde pancreatic drainage was attempted; however, it failed due to stenosis in the main pancreatic duct in the pancreatic body. Endoscopic ultrasound revealed a hypoechoic mass measuring 15 × 15 mm in the pancreatic body that was not enhanced in the late phase of contrast perfusion and was thus suspected to be an invasive ductal carcinoma. The patient underwent distal pancreatectomy with splenectomy and the postoperative course was uneventful. Histopathological examination confirmed a neuroendocrine tumor of the pancreas (NET G2). The main pancreatic duct was compressed by the tumor. Increased pressure on the distal pancreatic duct by the tumor might have caused formation of the pseudocyst and pleural effusion. To the best of our knowledge, this is the first case report of pancreatic pleural effusion associated with a neuroendocrine tumor.

**Conclusions:**

Differential diagnosis of a pancreatic neoplasm should be considered, especially when a patient without a history of pancreatitis presents with pleural effusion.

## Background

Pancreatic pleural effusion and ascites are defined as fluid accumulation in the thoracic and abdominal cavity, respectively, due to direct leakage of the pancreatic juice [1]. Pancreatic pleural effusion usually occurs in patients with acute or chronic pancreatitis. In chronic pancreatitis, various local complications such as pancreatic pseudocyst, biliary stenosis, pseudoaneurysm, and internal pancreatic fistula with ascites and pleural effusion can occur. Among these, the incidence of pancreatic pleural effusion has been reported to be as low as 0.4% [2]. Moreover, pancreatic pleural effusion associated with pancreatic neoplasm is rarer. Pancreatic neuroendocrine tumors (PNET) account for 1–5% of all pancreatic tumors and have a relatively favorable prognosis compared to pancreatic ductal adenocarcinoma [3]. The prevalence and incidence were reported to be 2.69 and 1.27, respectively, per 100,000 population; however, the number of patients with PNET has been increasing in Japan [4]. Here we report an extremely rare case of PNET with stenosis of the main pancreatic duct (MPD) that led to pancreatic pleural effusion.

## Case presentation

**Fig. 1:**
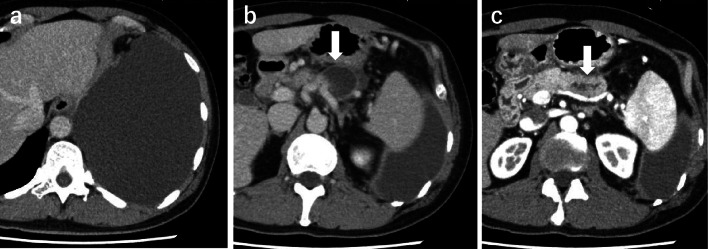
Findings of computed tomography (CT). CT reveals left-sided pleural effusion (**a**). Pancreatic pleural effusion was diagnosed on pleural puncture, based on the high amylase content (36,854 IU/L) in the pleural fluid. **b** CT shows a pseudocyst (arrow). **c** Dilation of the main pancreatic duct (arrow) in the pancreatic tail. No tumor can be observed in the pancreas

**Fig. 2:**
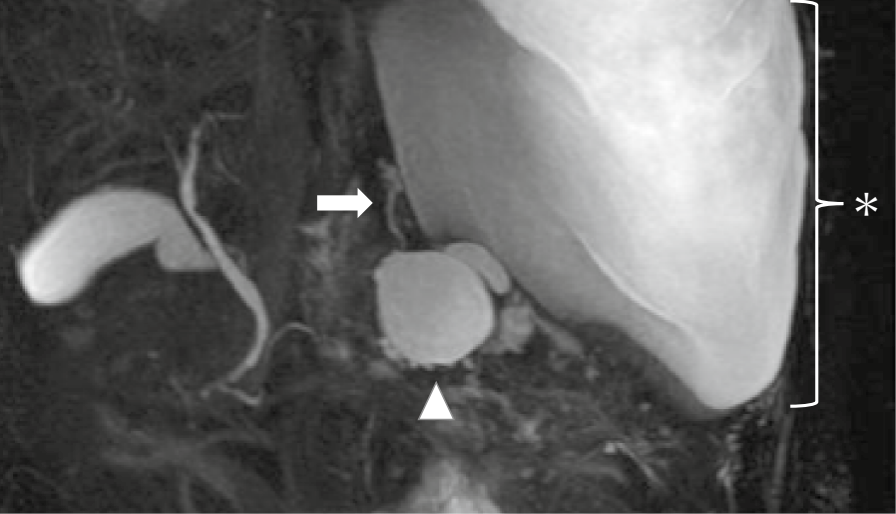
Findings of magnetic resonance cholangiopancreatography (MRCP). MRCP shows a fistula (arrow) from the pseudocyst (arrowhead) into the thoracic cavity (asterisk)

**Fig. 3:**
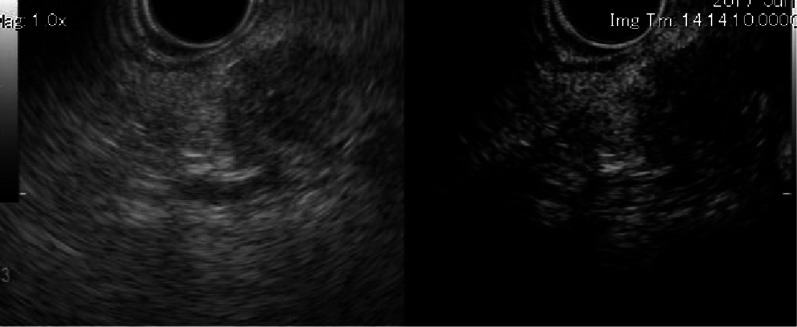
Findings of endoscopic ultrasound (EUS). **a** EUS reveals a hypoechoic mass measuring 15 × 15 mm in the pancreatic body. **b** The tumor shows hypoenhancement in the late phase of contrast EUS

A 51-year-old man complained of dyspnea. He was a non-smoker and did not consume alcohol, and there was no history of trauma or pancreatitis. Laboratory testing did not show any increase in IgG4 or tumor marker levels. A massive left-sided pleural effusion was detected on chest X-ray. On pleural puncture, it was diagnosed as pancreatic pleural effusion based on the high amylase content of 36,854 IU/L, at a previous hospital. Computed tomography (CT) scan showed a 35 mm pseudocyst and dilation of the MPD in the pancreatic tail, although no tumor mass in the pancreas or fistula into the thoracic cavity was detected (Fig. 1). However, magnetic resonance cholangiopancreatography (MRCP) showed a fistula opening into the left thoracic cavity (Fig. 2). Endoscopic retrograde cholangiopancreatography revealed that the MPD was stenosed in the pancreatic body and dilated in the pancreatic tail. Endoscopic nasopancreatic drainage (ENPD) was performed; however, it was ineffective, because the ENPD tube could not pass through the stenosis in the MPD. Infection and empyema occurred after the ENPD attempt, and these events could not be controlled via the chest tube. Eventually, thoracoscopic pleural resection was performed, following which the infection resolved. On the 50th day, he was referred to our hospital for further investigation and treatment. Examination revealed a small amount of discharge from the chest tube. Endoscopic ultrasound (EUS) revealed a hypoechoic mass measuring 15 × 15 mm in the pancreatic body, with calcification. The tumor was not enhanced in the late phase of contrast perfusion, and an invasive ductal carcinoma (IDC) was suspected (Fig. 3). We presumed that increased pressure on the distal pancreatic duct due to obstruction of the MPD caused by the IDC led to the formation of the pseudocyst, pancreatic fistula, and pleural effusion. We did not perform EUS-fine needle aspiration (EUS-FNA), because our preoperative diagnosis based on the imaging findings of EUS was pancreatic cancer, and the patient required distal pancreatectomy for management of the pancreatic pleural effusion as well as the pancreatic tumor. The results of EUS-FNA would not change the treatment plan; moreover, EUS-FNA could cause complications such as bleeding, pancreatitis, and seeding. On the 57th day, we performed distal pancreatectomy with splenectomy and removed the chest tube after the surgery. Intraoperatively, no liver metastasis, peritoneal dissemination, or ascites was observed. Tunneling behind the pancreas was performed at the level of the superior mesenteric vein. Intraoperative ultrasound showed the tumor in the pancreatic body and dilation of the MPD, and the pseudocyst on the left side of tumor. The pancreas was transected on the right side of the tumor, and the splenic artery was ligated at its root. Because of inflammation, there was severe adhesion around the pseudocyst behind the stomach. The serosa and muscle layers of the stomach had to be partially removed. The fistula could not be confirmed during the surgery. The intraoperative blood loss and operation time were 1755 mL and 188 min, respectively. The postoperative course was uneventful, and the patient was discharged on the 14th day after surgery.

**Fig. 4:**
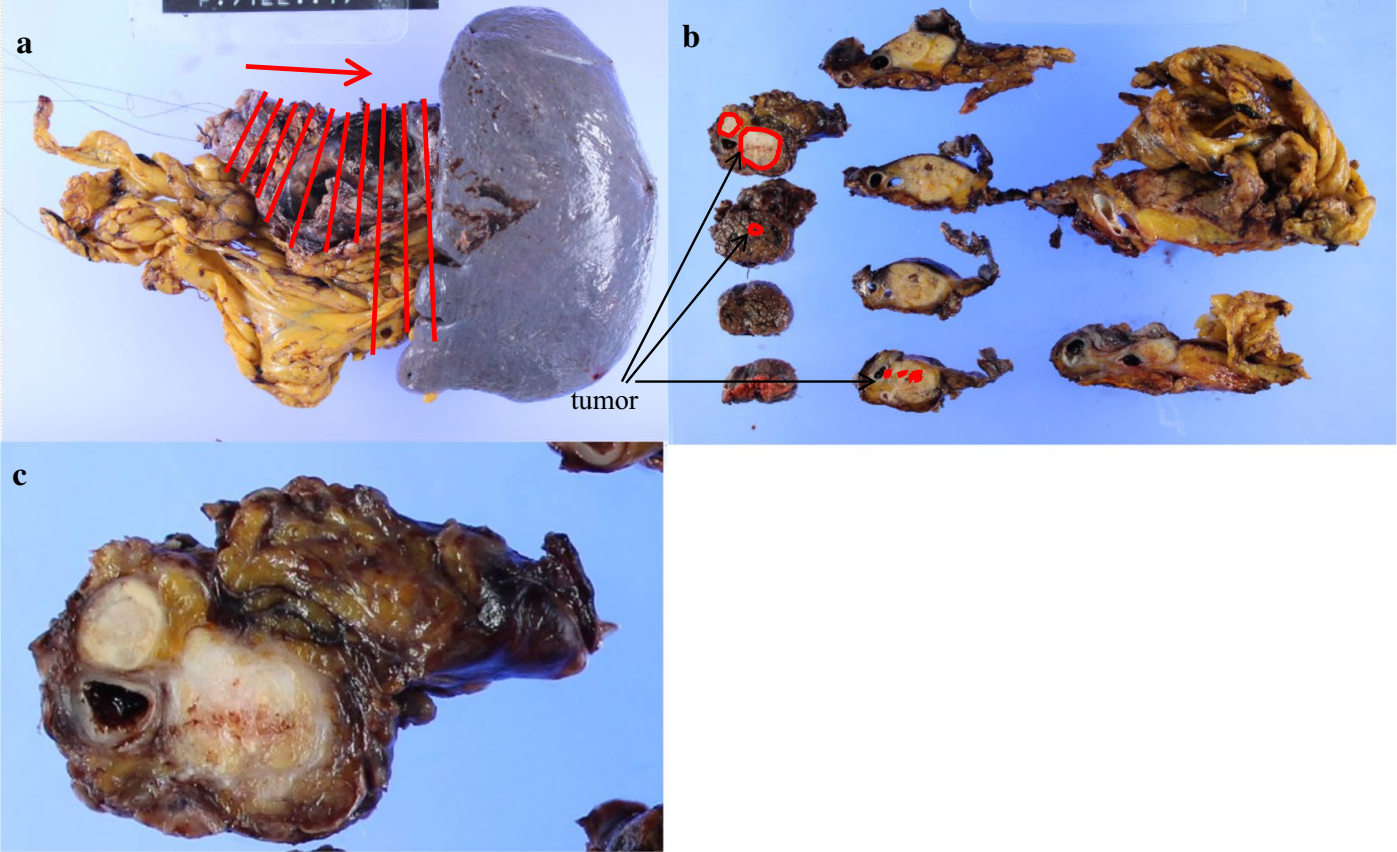
Resected specimen. **a** The resected specimen. Cutting lines of the resected specimen are shown (lines). **b** Gross description. The tumor cells represented in the circled area. **c** Solid tumors are seen in the macroscopic specimen

**Fig. 5:**
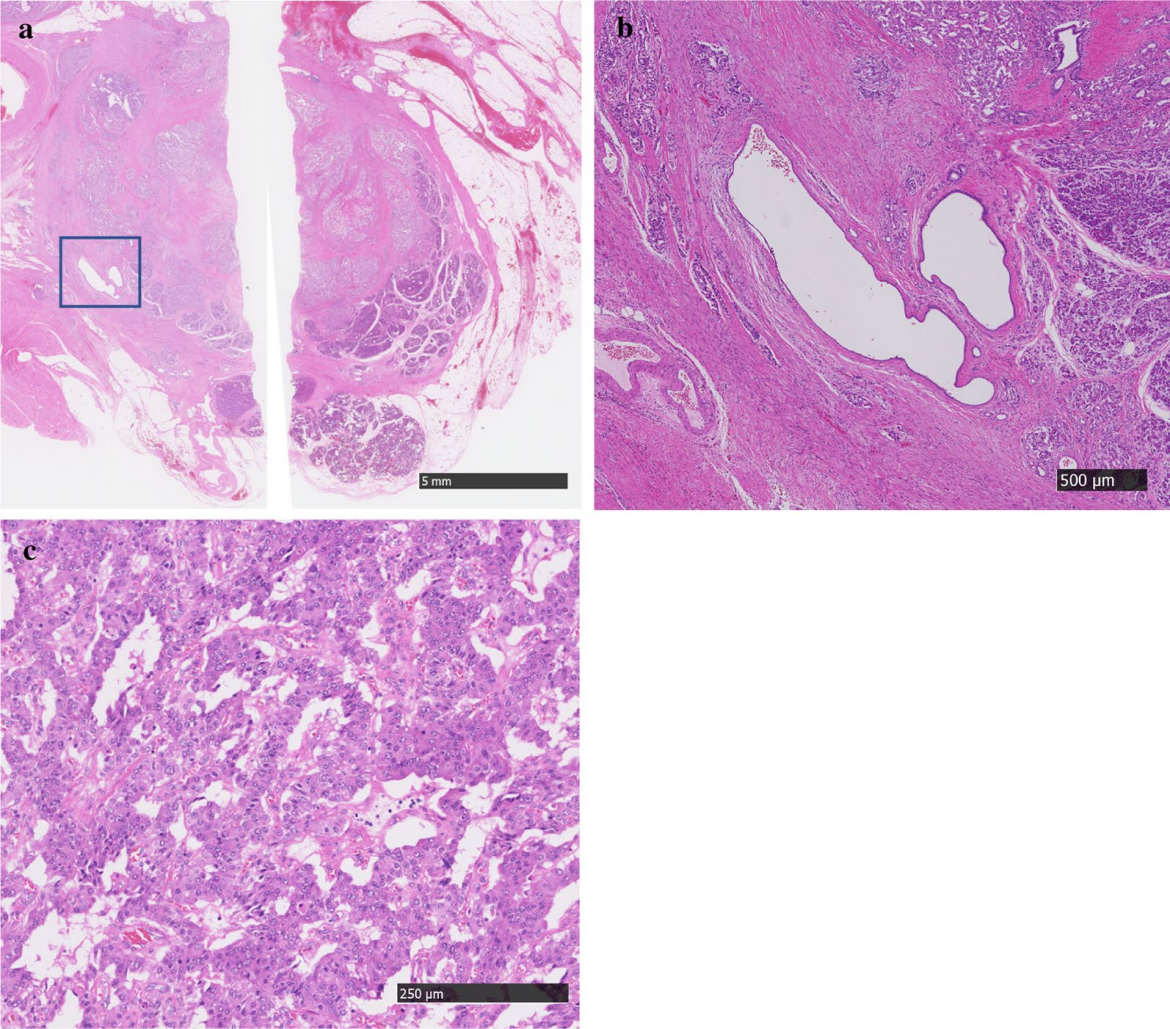
Findings of histopathological analysis. The tumor appears as a solid neoplastic lesion covered with a fibrotic capsule, and it measures 19 × 17 × 14 mm. Hematoxylin and eosin (HE) staining shows fibrosis. **a** HE, low-power microscopic view. The main pancreatic duct (square) is compressed by the tumor and narrowed. **b** HE, high-power microscopic view. In the tumor, eosinophilic cells show a ribbon-like arrangement. **c** Each cell shows swollen nuclei, anisonucleosis, and atypia. There were 9 mitoses observed in 10 high-power fields

**Fig. 6:**
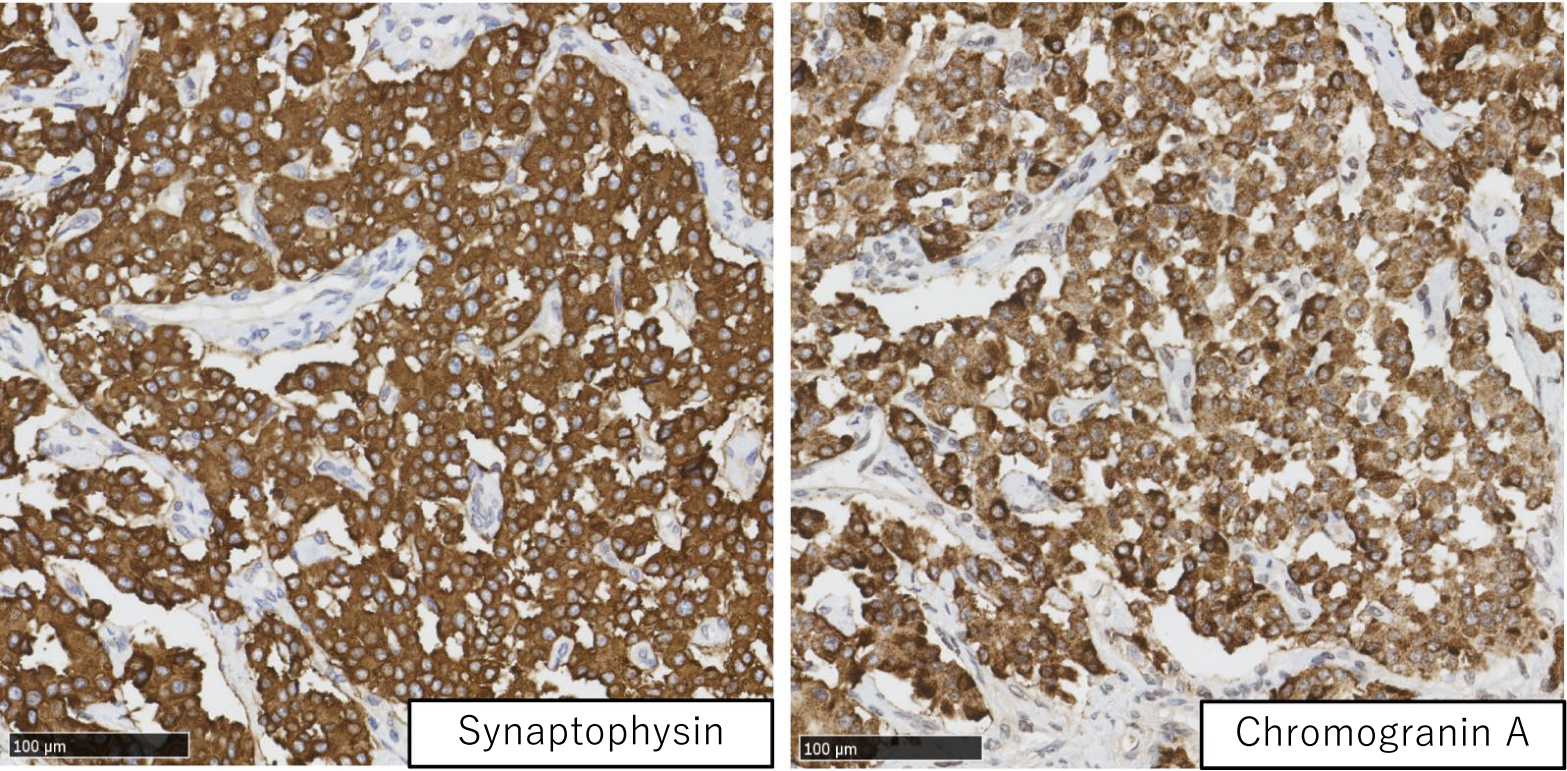
Findings of immunohistochemistry. Immunostaining shows positivity for chromogranin A and synaptophysin

Figure 4 shows the resected specimen, and Fig. 5 shows the findings of histopathological analysis. The tumor was a solid neoplastic lesion covered with a fibrotic capsule, and it measured 19 × 17 × 14 mm. Hematoxylin and eosin staining revealed fibrosis in the tumor. The MPD was compressed and narrowed by the tumor, and eosinophilic cells in the tumor showed a ribbon-like arrangement. Immunostaining revealed positivity for chromogranin A and synaptophysin and a Ki-67 index of 7.9% (Fig. 6). The final diagnosis was confirmed as neuroendocrine tumor (NET, G2). There was no metastasis in the lymph nodes. The patient was followed for 33 months after surgery without recurrence.

## Discussion

Pancreatic pleural effusion was termed as internal pancreatic fistula by Cameron et al. [1]. It usually occurs in patients with chronic pancreatitis but is rarely encountered in clinical practice. The reported incidence is 0.4% in patients with pancreatitis [2]. Most patients with pancreatic pleural effusion are men (90%), with a habit of alcohol consumption, and present with dyspnea and chest pain [5]. Gluck et al. [6] reported that the causes of pancreatic pleural effusion include rupture of a pancreatic pseudocyst (60%) and of pancreatic ducts due to chronic pancreatitis (15%) and pancreatic trauma (10%).

To the best of our knowledge, this is the first report of pancreatic pleural effusion associated with neuroendocrine tumor. However, there are 8 previous reports associated with neoplasms including IDCs. Table 1 shows the summary of the reported cases of pancreatic pleural effusion associated with pancreatic neoplasm, including our case [7–14]. These included 7 men and 2 women with a median age of 63 (37–70) years. Seven (78%) of 9 cases complained of dyspnea. In 4 cases (44%) including our case, a pancreatic fistula was detected on preoperative imaging studies. In 2 of these including our case, the fistula was detected by MRCP, preoperatively. Although Endoscopic retrograde cholangiopancreatography (ERCP) is useful in both diagnosing and managing a pancreatic fistula, the rate of identification of a fistula by ERCP is relatively low at 50% [15]. However, the rate of identification of a fistula on MRCP is reported to be 83% [16].

**Table 1:** Reported cases of pancreatic pleural effusion associated with pancreatic neoplasm

No	Author	Year	Age	Sex	Symptom	Tumor size (mm)	Tumor location	Pseudocyst	Stenosis of the MPD	Preoperative diagnosis of fistula	Fluid amylase (IU/l)	Treatment	Histological type	Prognosis (month)
1	Sankarankutty et al. [5]	1978	40	M	Dyspnea orthopnea	60	Pb	No	N/A	No	19,800	TP	IDC	8 alive
2	Kuroda et al. [6]	1983	37	F	Right back pain	150	Pb	Yes	No	No	975	DP	Mucinous cystadenocarcinoma	N/A
3	England et al. [7]	1988	64	M	Epigastric pain Vomitting	12	Pb	No	N/A	No	9600	None	oncocytic carcinoma	0.8 death
4	Shimaoka et al. [8]	2003	70	M	Dyspnea	12	Pb	No	Yes	No	22,665	DP	IDC	60 alive
5	Sugiyama et al. [9]	2010	63	M	Dyspnea Back pain	20	Ph	Yes	Yes	Yes	30,994	PpPD	IDC	13 alive
6	Cushen et al. [10]	2012	67	F	Dyspnea Chest pain	N/A	Pt	Yes	N/A	Yes	1,716,000	DP	IPMN Low-grade dysplasia	18 alive
7	Miyamoto et al. [11]	2017	62	M	Dyspnea Back pain	10	Pb	Yes	Yes	Yes	26,775	PD	IDC	19 alive
8	Saito et al. [12]	2020	69	M	Dyspnea	15	Pt	No	Yes	No	26,229	DP + partial gastrectomy	IDC	35 death
9	Our case	2020	51	M	Dyspnea	19	Pt	Yes	Yes	Yes	36,854	DP	PNET G2	33 alive

*MPD* main pancreatic duct, *Pb* pancreatic body, *N/A* not available, *TP* total pancreatectomy, *IDC* invasive ductal carcinomas, *DP* distal pancreatectomy, *Ph* pancreatic head, *PpPD* pylorus-preserving pancreatoduodenectomy, *Pt* pancreatic tail, *IPMN* intraductal papillary mucinous neoplasm, *PNET G2* pancreatic neuroendocrine tumor grade 2

In our case, the fistula into the left pleural cavity could not be identified intraoperatively and pathologically. However, based on imaging studies, especially MRCP, we presumed that increased pressure on the distal pancreatic duct due to the obstruction of the MPD by the tumor might have led to the development of a pseudocyst, pancreatic fistula, and pleural effusion. In fact, 5 (83%) of the 6 reported cases in Table 1 show stenosis of the MPD. The mechanism of development of a pancreatic pleural effusion in patients with chronic pancreatitis is similar. Stenosis of the MPD and dilation of the distal MPD are often seen in patients with chronic pancreatitis who develop a pancreatic pleural effusion [5]. It is well known that stenosis of the MPD is seen in patients with IDC, but it is uncommon in other neoplasms. In Table 1, IDC was the most commonly reported neoplasm in 6 patients (67%) with stenosis of the MPD in 4 of them. Additionally, Shi et al. reported that serotonin produced by PNET may be associated with local fibrosis and stenosis of the MPD [17]. However, the tumor in the present case showed negativity for serotonin on immunostaining (data not shown).

In our case, there were 2 imaging features atypical for PNET. First, the tumor was not visualized as a hyper-attenuating mass in the arterial phase and was not detected on contrast-enhanced CT. This might be associated with the high degree of fibrosis. The tumor was covered by a fibrotic capsule, and fibrosis was also observed within the tumor (Fig. 5a); these findings may be responsible for hypoenhancement of the tumor in the late phase of contrast-enhanced CT and EUS. It has been reported that fibrotic changes in the tumor are associated with a poor prognosis. Hypovascular PNET on CT images has been reported to have a high risk of recurrence [18]. There are several reports linking to worse biological features, such as higher tumor cell proliferation rate and poor postoperative survival [19]. Second, the MPD was involved by the tumor as described above. Recently, Nanno et al. [20] reported that MPD involvement was observed in 13 (13%) of 101 patients with well-differentiated PNETs. They also reported that on multivariate analysis, MPD involvement was significantly associated with nodal metastasis and recurrence. In general, small PNETs (≤ 2 cm) are known to have better outcomes, especially those that are asymptomatic and incidentally discovered [21]. However, it is also reported that symptomatic and small nonfunctional PNETs (≤ 2 cm) cause obstruction of the bile and/or pancreatic duct and have poor outcomes [22]. Therefore, our case might have a potential risk of an aggressive clinical course due to atypical tumor enhancement, MPD involvement, and presence of symptoms. In fact, although there was no metastasis to the lymph nodes, venous and nerve involvements were seen on histopathology. The patient needs to be followed up carefully.

## Conclusions

This was an extremely rare case of PNET with stenosis of MPD leading to pancreatic pleural effusion. Differential diagnosis of a pancreatic neoplasm should be considered, especially when any patient without a history of pancreatitis presents with pleural effusion.

## Data Availability

All datasets supporting the conclusions of this article are included within the article.

## Abbreviations

CT: Computed tomography
PNET: Pancreatic neuroendocrine tumor
MPD: Main pancreatic duct
MRCP: Magnetic resonance cholangiopancreatography
ENPD: Endoscopic nasopancreatic drainage
EUS: Endoscopic ultrasound
IDC: Invasive ductal carcinoma
EUS-FNA: Endoscopic ultrasound-fine needle aspiration
NET: Neuroendocrine tumor
ERCP: Endoscopic retrograde cholangiopancreatography
